# mSphere of Influence: Viruses—Pathogens or Expert Cell Biologists?

**DOI:** 10.1128/mSphere.00241-21

**Published:** 2021-03-31

**Authors:** Chelsey C. Spriggs

**Affiliations:** a Department of Cell & Developmental Biology, University of Michigan Medical School, Ann Arbor, Michigan, USA

**Keywords:** HCMV, host-pathogen interactions, virus

## Abstract

Chelsey C. Spriggs works in the field of DNA viral entry with a specific interest in virus-host interactions. In this mSphere of Influence article, she reflects on how two papers, “The HCMV assembly compartment is a dynamic Golgi-derived MTOC that controls nuclear rotation and virus spread” (D. J. Procter, A. Banerjee, M. Nukui, K. Kruse, et al., Dev Cell 45:83–100.e7, 2018, https://doi.org/10.1016/j.devcel.2018.03.010) and “Cytoplasmic control of intranuclear polarity by human cytomegalovirus” (D. J. Procter, C. Furey, A. G. Garza-Gongora, S. T. Kosak, D. Walsh, Nature 587:109–114, 2020, https://doi.org/10.1038/s41586-020-2714-x), impacted her research by reinforcing the scientific value in using viruses to understand cell biology.

## COMMENTARY

In the midst of this global pandemic, viruses are at the forefront of everyone’s mind—from scientific experts to the general public. Researchers of all fields have pivoted to study SARS-CoV-2 infection in hopes of learning how to limit its spread and put an end to the devastation that it has caused over the past year. The value in virology research is now clearer than ever, but aside from the very important goal of preventing and curing virus-related disease, studying virus infection has much broader implications for human health. Viruses are obligate intracellular parasites, meaning that they cannot replicate or survive outside a host. This also means that, from the moment that they infect cells, they provide opportunities for us to learn not only about their pathogenesis but also about the biology of the cells that they infect. As viruses have evolved with their hosts for thousands of years, examining how they either utilize or avoid cellular factors to promote infection has often been the basis of new scientific knowledge and discovery. In these two papers, Procter et al. demonstrate just that through their investigation of how another important human pathogen, human cytomegalovirus (HCMV), manipulates the cell during infection (1, 2).

HCMV is a leading cause of congenital birth defects worldwide, and with no vaccine or cure available, understanding the biology of its infection may lead to new drug targets and treatment options. During infection, HCMV forms a unique virion assembly compartment (AC) in the cytoplasm into which virus particles bud from the nucleus to mature. While previous studies had shown that AC formation results from the reorganization of the Golgi membrane (3), Procter et al. (1) used live-cell imaging to further characterize the dynamic nature of this process. The authors’ major findings revealed that the virus-induced AC also acts as a Golgi-derived microtubule organizing center (MTOC) and that its formation requires the action of the MT plus-end-binding protein EB3, but not EB1. This diverges from a recent report that Golgi membrane-based MT formation uses both EB1 and EB3 (4), possibly highlighting novel functional differences between these cellular proteins and identifying a new target for suppressing virus infection. Importantly, the authors’ innovative use of live-cell imaging led to the significant observation that nuclei in infected cells exhibit persistent rotation that is not observed in uninfected cells. Precisely how and why HCMV induces nuclear rotation would become the basis of their next paper.

In order to further investigate this virus-induced phenomenon, Procter et al. (2) developed convolutional neural network (CNN)-based automated cell classification and analysis pipelines to measure the spatial organization of thousands of individual cells. The authors found that acetylated MTs generated at the viral AC interact with linker of nucleoskeleton and cytoskeleton (LINC) complexes in the nuclear membrane to rotate the nucleus through cytoplasmic dynein motor binding domains in Nesprin-2. This polarizes the nuclear membrane, drawing inactive histones and associated host cellular DNA toward the AC and segregating viral DNA to create an optimal environment for virus replication. In addition, this study provides new insight into the role of acetylated MTs, LINC complexes, and the nucleoskeleton in nuclear positioning and polarity during normal cellular processes, including cell migration and meiosis (5, 6).

These two papers exemplify the dual scientific potential of studying virus-host interactions. In researching how to prevent infection by a medically relevant human pathogen, Procter et al. not only identified a potential drug target for preventing HCMV infection but also contributed to our general knowledge of the nuclear positioning mechanisms that are often disrupted in other disorders, such as lissencephaly and myopathy (7). What really captivated me, however, is that their fascinating discovery that HCMV induces nuclear rotation to control intranuclear polarity during viral replication may not have occurred had they not decided to use live-cell imaging to answer a different research question. As scientists, we are trained to perform hypothesis-driven research, but this is an excellent reminder that observation-based science and technical innovation can be equally beneficial to scientific advancement. In fact, some of the best hypotheses are forged from the most perplexing and unexpected observations.

As a postdoctoral fellow, I research the virus-host interactions required for entry of the simian virus 40 (SV40) polyomavirus (PyV). Although SV40 does not cause disease in humans, it has been used both to elucidate the biology of human PyV infection and as a valuable tool in cell biology. Because SV40 relies largely on cellular components to complete its life cycle, studies of SV40 DNA synthesis proved instrumental in dissecting the mechanisms of eukaryotic DNA replication. Moreover, the first nuclear localization sequence was identified in SV40, paving the way for future studies on nucleocytoplasmic transport for years to come. Our lab combines classical biochemistry with more modern approaches to examine how SV40 reaches the nucleus to establish infection as well as to investigate various aspects of cell biology. By reconstituting SV40 disassembly *in vitro* for the first time, we recently identified a surprising role for dynein cargo adaptors in viral uncoating that is independent of the dynein motor itself (8). This unanticipated result suggests that cargo remodeling may be a novel function of these host proteins, and it would be interesting to determine normal cellular processes in which it is used. As for my future research, I have been inspired by the authors to continue to incorporate innovative strategies into the field of virus-host interactions while remembering to chase the unexpected.

As a society, our focus over the past year rightfully has been to understand the infection and pathogenesis of SARS-CoV-2 in hopes of ending the ongoing global health emergency. When this pandemic is over, however, and viruses are no longer on (some of) our minds, I urge you to consider the value in studying these viral pathogens—the expert cell biologists.
